# Isolation and characterisation of polymorphic microsatellite loci for studies of the big blue octopus, *Octopus cyanea*

**DOI:** 10.1007/s12526-017-0717-0

**Published:** 2017-04-29

**Authors:** Niall J. McKeown, Amy L. Taylor, Paul W. Shaw

**Affiliations:** 1 Institute of Biological, Environmental and Rural Sciences (IBERS), Aberystwyth University, Penglais Campus, Aberystwyth, SY23 3DA UK; 2 Royal Holloway University of London, Egham, Surrey TW20 0EX UK

**Keywords:** Marine invertebrate, Genetic, Biodiversity, Population genetics, Conservation, Management

## Abstract

The big blue octopus, *Octopus cyanea*, occurs on coral reefs throughout the Indo-Pacific region from East Africa to the Hawaiian Islands, wherein it is of great ecological and socio-economic importance. However, many components of its intraspecific biodiversity, such as population structure, are unresolved due to a lack of informative genetic markers. To address this issue, which may compromise conservation and sustainability efforts, the development and characterisation of the first species-specific microsatellite loci for *O. cyanea* are described here. The eight loci were characterised by the genotyping of 40 adults from Madagascar, which revealed an average of 13.5 alleles per locus (range 9–18). The observed and expected heterozygosity per locus ranged from 0.432 to 0.949 and from 0.481 to 0.989, respectively. No evidence of linkage disequilibrium was detected between pairs of loci. Genotype proportions at six loci conformed to Hardy–Weinberg equilibrium expectations, with two loci exhibiting significant heterozygote deficits. These loci are applicable to multiple areas of eco-evolutionary research and, thus, represent a valuable resource for future studies of *O. cyanea*.

*Octopus cyanea*, commonly referred to as either the big blue octopus or day octopus, is a benthic species occurring on coral reefs throughout the Indo-Pacific region from East Africa to the Hawaiian Islands. Artisanal fishing for *O. cyanea* is an important economic and subsistence activity along the eastern coast of Africa and islands of the Southwestern Indian Ocean (Guard and Mgaya 2002), providing a national source of foreign currency earnings through export to Europe and the Far East and a protein-rich food source for local people. Despite the socioeconomic importance of the species, and the increasing harvesting pressure, fishery data are extremely limited. Taylor et al. (2012) developed a genetic assay permitting the identification of *O. cyanea* and two co-occurring morphologically similar species (*O. vulgaris* and *Callistoctopus ornatus*) and, thus, offering potential for the collection of species-specific catch data and other information (e.g. McKeown et al. 2015) relevant to conservation and management. However, while *O. cyanea* is recognised as an important component of reef communities (Guard and Mgaya 2002), little is known about its intraspecific biocomplexity and informative genetic markers, such as microsatellites, would greatly enhance eco-evolutionary studies. This research reports the development of the first polymorphic microsatellite markers from *O. cyanea*.

Microsatellites were isolated from an enriched partial genomic library created by methods outlined by Glenn and Schable (2005) and McKeown and Shaw (2008). Briefly, genomic DNA extracted from muscle tissue was digested with the restriction enzyme *Rsa*I (New England Biolabs) and the blunt-ended fragments ligated to double-stranded SuperSNX linkers. Enrichment was then performed by selective hybridisation of biotin-labelled repeat motif oligonucleotide probes [(TG)_12_, (GA)_12_, (AAAT)_8_, (AACT)_8_, (AAGT)_8_, (ACAT)_8_, (AGAT)_8_] with hybridised complexes captured using streptavidin-coated magnetic beads (DYNAL) and unbound DNA removed by a series of washes. DNA fragments were then eluted from the magnetic beads and amplified by polymerase chain reaction (PCR) using the SuperSNX24F oligonucleotide. The PCR products were cloned using the TOPO-TA Cloning Kit (Invitrogen) and recombinant colonies identified by disruption of β-galactosidase activity. Recombinants were individually transferred into 50 μL of water and incubated at 95 °C for 10 min to promote plasmid DNA release. One microlitre of each plasmid extract was submitted to PCR using M13 forward and reverse primers. The PCR mixture contained 1× buffer, 1.5 mM MgCl_2_, 0.2 mM dNTPs, 0.2 U of *Taq* DNA polymerase (Bioline, UK), 1 pmol of each primer and the thermoprofile consisted of 30 cycles of 95 °C for 30 s, 50 °C for 30 s and 72 °C for 30 s. PCR products were then sequenced directly using the internal T7 vector primer. For sequences containing microsatellite arrays, PCR primers were designed from flanking regions using the program Primer3 (Untergasser et al. 2012).

Eight microsatellite loci (Table 1) were assessed by genotyping 40 adult *O. cyanea* collected from Madagascar. For each locus, the respective forward primer was labelled with a fluorescent dye at the 5′ end (Life Technologies). Each locus was individually PCR amplified in a 10-μL reaction containing 100–200 ng DNA, 5 μL Biomix (Bioline, UK) and 0.2 pmol of each primer. PCR thermoprofiles included an initial denaturation step (95 °C for 3 min), followed by 35 cycles of 95 °C for 30 s, 55 °C for 60 s and 72 °C for 30 s. Amplicons were separated using an AB3500 genetic analyser (Applied Biosystems) and alleles designated using the GeneMapper software (version 4.1, Applied Biosystems).

**Table 1:** Summary information for eight microsatellite loci developed for *Octopus cyanea*, including primer sequences, repeat motif observed in the clone used to develop each locus, allele numbers (*N*_a_), size range, observed (*H*_O_) and expected (*H*_E_) heterozygosity and *P*-values for tests of Hardy–Weinberg equilibrium (*P*_HW_) calculated from the analysis of 40 adults from Madagascan waters

Locus	Primer sequences (5′–3′)	Repeat motif in cloned allele	*N*_a_	Size range (bp)	*H*_O_	*H*_E_	*P*_HW_
Ocya-1	F: TTTCATCCACCCATTGATATCTC R: CATCATGGTGTCTGATATGGC	(GTAT)_13_	14	156–212	0.949	0.906	0.816
Ocya-2	F: TTCAACACTCAAATCGTTAGGG R: TGAACAGAGCTCTTAATTACTATGAA	(CATA)_17_	9	200–248	0.432	0.481	0.109
Ocya-3	F: TCCCGCATCAACCTTTAATG R: TTAGCCGGACCGTTAGTTTG	(GATA)_19_	16	208–272	0.872	0.925	0.767
Ocya-4	F: AAATGCACACGCATAAAAACA R: GCGTCAGGTCCAGGACTTAT	(CATA)_20_	14	172–224	0.850	0.989	0.128
Ocya-5	F: ACATCGACCCCAGGACTTAG R: TGCAATAACCTTCGGTTGTG	(CA)_7_…(CA)_6_…(CA)_7_…(CA)_5_	10	320–384	0.433	0.870	**<0.001**
Ocya-6	F: TAAGTCCTGGGATCGAATGG R: TGCCCCTTTAAAATAGAGTTGC	(GTAT)_14_	13	212–284	0.872	0.883	0.769
Ocya-7	F: TAGAATGTGCGTCGCAAAAG R: GCGTAAGCATGCATGTATGC	(CATA)_16_	18	128–196	0.949	0.942	0.679
Ocya-8	F: TTATGAAATTGGGTGGACGG R: ACATCGACTCACGTGCACAC	(GT)_6_…(GT)_15_	14	215–253	0.556	0.989	**<0.001**

All loci produced high quality products, with allele sizes differing in expected multiples of their repeated motifs. However, in the case of Ocya-5, alleles differed in multiples of four base pairs, despite the microsatellite array being composed of a dinucleotide motif. Standard diversity indices for each locus along with primer sequences and allele size ranges are presented in Table 1. Tests for linkage disequilibrium (LD) and deviations from Hardy–Weinberg equilibrium (HWE) expectations were performed using default parameters in GENEPOP 4.0 (Raymond and Rousset 1995). No significant LD was detected between any locus pair. Genotype proportions conformed to HWE expectations for 6 of the 8 loci, with Ocya-5 and Ocya-8 exhibiting significant heterozygote deficits. Such heterozygote deficits are quite common for cephalopod microsatellites (McKeown and Shaw 2014) and may be due to null alleles (Shaw et al. 2010). The markers described here represent the first microsatellites isolated from, and characterised in, *O. cyanea* and offer considerable potential as tools for diverse areas of eco-evolutionary research, such as studies of population structure/dynamics, phylogeography and adaptation. Cephalopods display some of the most complex behavioural adaptations among marine invertebrates, particularly in respect to their mating strategies (Hanlon and Messenger 1996). For *O. cyanea*, sexual cannibalism (Hanlon and Forsythe 2008) and putative sexual displays (Wells and Wells 1972) have been described and these microsatellites could be used to investigate such behaviours in the context of fertilisation success (e.g. Naud et al. 2016).
